# Machine learning based readmission and mortality prediction in heart failure patients

**DOI:** 10.1038/s41598-023-45925-3

**Published:** 2023-10-31

**Authors:** Maziar Sabouri, Ahmad Bitarafan Rajabi, Ghasem Hajianfar, Omid Gharibi, Mobin Mohebi, Atlas Haddadi Avval, Nasim Naderi, Isaac Shiri

**Affiliations:** 1 Department of Medical Physics, School of Medicine, Iran University of Medical Science, Tehran, Iran; 2 Rajaie Cardiovascular Medical and Research Center, Iran University of Medical Science, Tehran, Iran; 3 Echocardiography Research Center, Rajaie Cardiovascular Medical and Research Center, Iran University of Medical Sciences, Tehran, Iran; 4 Cardiovascular Interventional Research Center, Rajaie Cardiovascular Medical and Research Center, Iran University of Medical Sciences, Tehran, Iran; 5 Department of Biomedical Engineering, Tarbiat Modares University, Tehran, Iran; 6 School of Medicine, Mashhad University of Medical Sciences, Mashhad, Iran; 7 Division of Nuclear Medicine and Molecular Imaging, Geneva University Hospital, 1211 Geneva 4, Switzerland; 8 Department of Cardiology, Inselspital, Bern University Hospital, University of Bern, Bern, Switzerland

**Keywords:** Cardiology, Health care economics

## Abstract

This study intends to predict in-hospital and 6-month mortality, as well as 30-day and 90-day hospital readmission, using Machine Learning (ML) approach via conventional features. A total of 737 patients remained after applying the exclusion criteria to 1101 heart failure patients. Thirty-four conventional features were collected for each patient. First, the data were divided into train and test cohorts with a 70–30% ratio. Then train data were normalized using the Z-score method, and its mean and standard deviation were applied to the test data. Subsequently, Boruta, RFE, and MRMR feature selection methods were utilized to select more important features in the training set. In the next step, eight ML approaches were used for modeling. Next, hyperparameters were optimized using tenfold cross-validation and grid search in the train dataset. All model development steps (normalization, feature selection, and hyperparameter optimization) were performed on a train set without touching the hold-out test set. Then, bootstrapping was done 1000 times on the hold-out test data. Finally, the obtained results were evaluated using four metrics: area under the ROC curve (AUC), accuracy (ACC), specificity (SPE), and sensitivity (SEN). The RFE-LR (AUC: 0.91, ACC: 0.84, SPE: 0.84, SEN: 0.83) and Boruta-LR (AUC: 0.90, ACC: 0.85, SPE: 0.85, SEN: 0.83) models generated the best results in terms of in-hospital mortality. In terms of 30-day rehospitalization, Boruta-SVM (AUC: 0.73, ACC: 0.81, SPE: 0.85, SEN: 0.50) and MRMR-LR (AUC: 0.71, ACC: 0.68, SPE: 0.69, SEN: 0.63) models performed the best. The best model for 3-month rehospitalization was MRMR-KNN (AUC: 0.60, ACC: 0.63, SPE: 0.66, SEN: 0.53) and regarding 6-month mortality, the MRMR-LR (AUC: 0.61, ACC: 0.63, SPE: 0.44, SEN: 0.66) and MRMR-NB (AUC: 0.59, ACC: 0.61, SPE: 0.48, SEN: 0.63) models outperformed the others. Reliable models were developed in 30-day rehospitalization and in-hospital mortality using conventional features and ML techniques. Such models can effectively personalize treatment, decision-making, and wiser budget allocation. Obtained results in 3-month rehospitalization and 6-month mortality endpoints were not astonishing and further experiments with additional information are needed to fetch promising results in these endpoints.

## Introduction

Heart Failure (HF) is the underlying cause of over one-third of cardiovascular deaths, with more than 64 million sufferers worldwide^1^. Acute Heart Failure (AHF) is a clinical condition caused when the myocardium function is either lost or exacerbated rapidly or quickly. As a result of this condition, the heart is often unable to sustain a sufficient cardiac output and meet metabolic demands. This condition puts patients’ quality of life, function, and lifespan at risk^2,3^.

HF is a common disorder that can increase in-hospital mortality^4^. Furthermore, AHF has 30-day and 1-year hospital readmission rates of 16–19% and 53%, respectively, as depicted in various trials^5,6^. In Khan et al*.*^7^ study, 6,669,313 and 5,077,949 HF rehospitalizations cases for 30 and 90 days were examined, and 18.2% and 31.2% were readmitted in each group, respectively. In a study by Fudim et al*.*^8^, patients who were readmitted within 30 days were likelier to die (all-caused death) after six months. Furthermore, readmissions account for a significant fraction of overall healthcare expenditures, making them costly for public and private payers in any country^9^. In a study by Lahewala et al.^10^, 21.3% of 715,993 HF cases were readmitted to a different hospital. When they were referred to a different hospital, they encountered a higher rate of in-hospital mortality, length of stay, and expenses ($22,602 against $13,740). Beyond monetary costs, rehospitalization would expose patients to the potential dangers of stress and hospital-acquired infections^11^. Hospitals have enacted various strategies for reducing readmission rates, such as patient education and proper follow-up after discharge. The education provided to patients may include specific guidelines about when to return for follow-up appointments and how to utilize their medications properly. By empowering patients with this knowledge, they become more informed and better equipped to manage their condition at home, which can help to prevent future hospitalizations. Nevertheless, these approaches are highly time-consuming and labor-intensive. Hence, identifying factors associated with readmission seems crucial for better recognizing appropriate preventive methods^11–15^.

Several studies have been conducted to predict which patients will be readmitted, identifying multiple etiologies for AHF readmission^16^. Some factors, such as age, sex, and race, are tied to the patient, while others, like misdiagnosis and inadequate post-discharge follow-up, depend on the healthcare system. Such factors are giving rise to preventable readmissions^17^. Recently, an ongoing trend has been toward implementing Machine Learning (ML) techniques as predictive models for analyzing and classifying different data types. ML is a scientific discipline seeking to solve complex challenges to build models based on data sets containing significant information without explicit programming rules^18–21^.

In several in-hospital mortality studies using ML, the Area Under the ROC Curve (AUC) ranges from 0.720 to 0.913 were reported^22–24^. Many studies have also been conducted on 30-day readmission, with AUCs ranging from 0.546 to 0.784^25–34^. In two studies concerning 3-month readmission, the AUC value ranged from 0.570 to 0.770^31,35^. According to Shin et al.^33^, ML methods outperform conventional statistical models in predicting HF readmissions and mortalities. Using conventional features, our study exerts ML algorithms to predict in-hospital and 6-month mortality, as well as 30-day and 3-month hospital readmission rates.

## Material and methods

**Figure 1:**
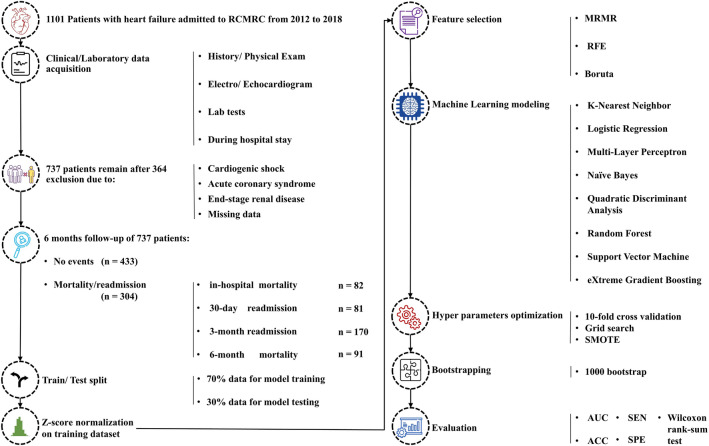
A visual demonstration of all the steps involved in this study.

Figure 1 illustrates a summary of the steps taken in the current study. Each stage will be fully reviewed in the following sections.

### Patients’ data

In the present study, we used the data from the Rajaie Acute Systolic Heart Failure (RASHF) registry^36^. RASHF is a prospective hospital-based study conducted at Rajaie Cardiovascular Medical and Research Center (RCMRC). Observations were made on 1101 hospitalized systolic HF patients with acute decompensation before detecting any endpoints. This study has been approved by the Iran University of Medical Sciences (IUMS). All protocols and methodologies employed adhered to ethical guidelines. Patient consent procedures were exempted by the IUMS ethics committee.

The present prospective work includes adult patients with Left Ventricle Ejection Fraction (LVEF) < 40% and either newly developed or deteriorating HF symptoms. Patients’ data has been collected based on international definitions and guidelines over a period of 6 years^37^. Of 1101 patients, 364 were excluded due to missing data or suffering from cardiogenic shock, acute coronary syndrome, and chronic hemodialysis for end-stage renal disease. Such patients undergo different management compared to other AHF patients. In total, 737 patients were included in this study, among which 529 patients were men (71.8%, mean age = 55.00) and 208 were women (28.2%, mean age = 57.89).

An experienced registry team collected the data on admission day and during the hospital stay^36^. A detailed history was collected on the first day of the hospital stay, and an experienced cardiologist performed a thorough physical examination and echocardiography. The cardiologist measured LVEF, Pulmonary Artery Pressure (PAP), Inferior Vena Cava (IVC) size, Right Ventricular (RV) dysfunction, Mitral Regurgitation (MR), and Tricuspid Regurgitation (TR) in echocardiography. After examining the electrocardiogram, they also reported the wide QRS complex and atrial fibrillation (AF) parameters. In addition, laboratory data such as sugar level, Complete Blood Count (CBC), serum creatinine (Cr), Blood Urea Nitrogen (BUN), potassium (K), sodium (Na), magnesium (Mg), bilirubin, and liver enzymes were collected on admission day. These tests were done using conventional standard laboratory procedures in Rajaie Hospital’s laboratory. BUN and Cr levels were measured daily until the discharge day. Thirty-four features and four considered endpoints are depicted in Table 1 with their explanation. Values of these features can also be seen in Table 1S*.* The features used in the study were derived from guidelines related to heart failure patients, which are established and accepted medical standards for the assessment and treatment of this condition. These guidelines include recommendations for specific diagnostic tests, laboratory values, or other clinical measures that are relevant to the management of heart failure^37,38^. Regarding the endpoints, they are the most frequent endpoint both in the clinic and in recent studies^36,39^.

**Table 1:** The general definition of all features.

		Column name	Definition	Description
Binary Features	History/physical Exam	Diabetes	Presence of diabetes mellitus	Yes/no
		Hypertension	Presence of hypertension	Yes**/**no
		IHD/CAD	Presence of ischemic heart disease or coronary artery disease	Yes**/**no
		CRF	Presence of chronic kidney failure	Yes**/**no
		Smoking history	History of smoking	Yes**/**no
		ICD**/**CRT	Presence of intracardiac defibrillator or cardiac resynchronization therapy	Yes**/**no
		Edema	Presence of Edema	Yes**/**no
		Ascites	Presence of ascites	Yes**/**no
		Infection	Presence of infection on admission	Yes**/**no
	Electro/Echocardiogram	Wide QRS complex	Presence of wide QRS in ECG on admission	Yes**/**no
		AF	Presence of atrial fibrillation rhythm on admission	Yes**/**no
		RV dysfunction*	At least moderate right ventricular dysfunction severity by echo	Yes**/**no
		MR*	At least moderate mitral regurgitation by echo	Yes**/**no
		TR*	At least moderate tricuspid valve regurgitation by echo	Yes**/**no
	During hospital stay	Abnormal LFT	Presence of abnormal liver function test during admission	Yes**/**no
		WRF**	Presence of worsening renal function during admission	Yes**/**no
		Dialysis	Performing dialysis for a patient during admission	Yes**/**no
		Inotrope	Using inotrope drugs for patients during admission	Yes**/**no
	Follow-up	In-hospital mortality	In-hospital death	Yes**/**no
		3-month readmission	Rehospitalization within three months	Yes**/**no
		1-month readmission	Rehospitalization within one month	Yes**/**no
		6-month mortality	Discharge but dead in 6 months	Yes**/**no
Other Features	History/physical Exam	Sex (male, female)	The patient’s gender	Male**/**female
		Age	The patient’s age	Years
		HF etiology	Cause of heart failure	Ischemic**/**non-ischemic
		HR	Heart Rate	Heartbeats per minute
		SBP	Systolic blood pressure	Systolic blood pressure by millimeter of mercury
		NYHA Class	New York heart association class (the degree of dyspnea)	1–2–3–4 (ordinal)
	Electro/Echocardiogram	LVEF (%)	Left ventricular ejection fraction by echo	percent (%)
		PAP	Pulmonary artery pressure by echo	in mmHg
		IVC size	Inferior vena cava size	millimeters
	Lab tests	Cr	Baseline (first) serum creatinine level	mg/dl
		BUN	Baseline BUN level	mg/dl
		Uric acid	Baseline serum uric acid level	mg/dl
		Hb	Baseline Hemoglobin level	d/dl
		Na	Baseline serum sodium	mg/dl
		Pro-BNP	Baseline Pro BNP level	pg/mL
		Discharge Cr	Serum creatinine level at the discharge or death day	mg/dl

*HF* heart failure, *BUN* blood urea nitrogen, *CRF* chronic renal failure, *Cr* serum creatinine, *Hb* hemoglobin, *ICD* implantable cardioverter defibrillator, *CRT* cardiac resynchronization therapy, *IHD* ischemic heart disease, *CAD* coronary artery disease, *IVC* inferior vena cava, *LFT* liver function tests, *LVEF* left ventricular ejection fraction, *MR* mitral valve regurgitation, *Na* serum sodium, *NYHA* New York heart associatioَn, *PAP* pulmonary artery pressure, *Pro BNP* pro brain natriuretic peptide, *RV* right ventricle, *SBP* systolic blood pressure, *TR* tricuspid valve regurgitation, *AF* atrial fibrillation, *WRF* worsening renal function.

*Should be at least moderate, according to the experienced cardiologist’s echocardiography report.

**During the index hospitalization up until discharge, WRF (worsening renal function) was defined as a definite rise in serum creatinine of 0.3 mg/dL from baseline (on the first admission day).

After discharge, all patients were monitored from hospital documents and/or phone calls for up to six months to be followed up on mortality and readmission. Predictions of patient outcomes were evaluated under four specific criteria: (1) patients who died in the hospital (in-hospital mortality), (2) patients who died within six months (6-month mortality), (3) patients who were readmitted within 30 days (30-day hospital readmission), and (4) patients who were readmitted within 90 days (3-month hospital readmission). Among 737 patients, 433 had none of these conditions; however, 304 patients experienced at least one event: 82 in-hospital mortality, 81 30-day hospital readmission, 170 3-month hospital readmission, and 91 6-month mortality.

### Train-test split

A 70:30 ratio was used to split the dataset into training and testing groups for each individual endpoint. First, the data underwent a comprehensive review to deal with missing and outlier data. Data that deviated by more than three standard deviations from the mean were omitted as outliers. Continuous missing variables were imputed using averaging, and categorical features with missing values were excluded from the analysis. Then, train data were normalized using the Z-score method, and its mean and standard deviation were applied to the test data. In the in-hospital mortality endpoint, all the samples of this study were used, but for the other three endpoints, the patients who died in the hospital were excluded.

### Feature selection and ML model development

Three feature selection methods were used to select the important features: (1) Recursive Feature Elimination (RFE), (2) Minimum Redundancy Maximum Relevant (MRMR), and (3) Boruta (detailed explanations of each method can be found in the Supplementary Material S1). Next, the ML modeling was performed using eight algorithms, including K-Nearest Neighbor (KNN), Logistic Regression (LR), Multi-Layer Perceptron (MLP), Naïve Bayes (NB), Quadratic Discriminant Analysis (QDA), Random Forest (RF), Support Vector Machine (SVM), and eXtreme Gradient Boosting (XGB).

Next, hyperparameter optimization was performed using stratified tenfold cross-validation (to minimize overfitting) and grid search in the training dataset. Then, the Synthetic Minority Oversampling Technique (SMOTE) was used to address the class imbalance issue in our data during cross-validation and hyperparameter optimization in the training set. While tenfold cross-validation was used, nine folds were oversampled with SMOTE, and one fold remained as the original validation set. Then, after finding the optimal hyperparameters, SMOTE was applied to the entire training data and models with optimum hyperparameter were trained with this dataset. At last, 1000 bootstraps were executed on the test dataset. Therefore, three feature selection methods and eight ML models bring 24 models for each of the four mentioned endpoints.

### Model evaluation and statistical analysis

The 24 developed models were evaluated using four metrics: area under the receiver operating characteristic curve (AUC), accuracy (ACC), sensitivity (SEN), and specificity (SPE). The AUCs of all models were compared using the Wilcoxon rank sum test, and the False Discovery Rate (FDR) was then corrected using the Benjamini–Hochberg technique. As a result, modified p-values, also referred to as q-values were evaluated. P-values under 0.05 were regarded as statistically significant. All models were developed in R programming language 4.0 using mlr library 2.18^40^ in Ubuntu 18.04.

### Ethics approval

This retrospective study was approved by the ethics committee of Iran University of Medical Sciences (IR.IUMS.FMD.REC.1398.404).

## Results

### Feature selection

**Figure 2:**
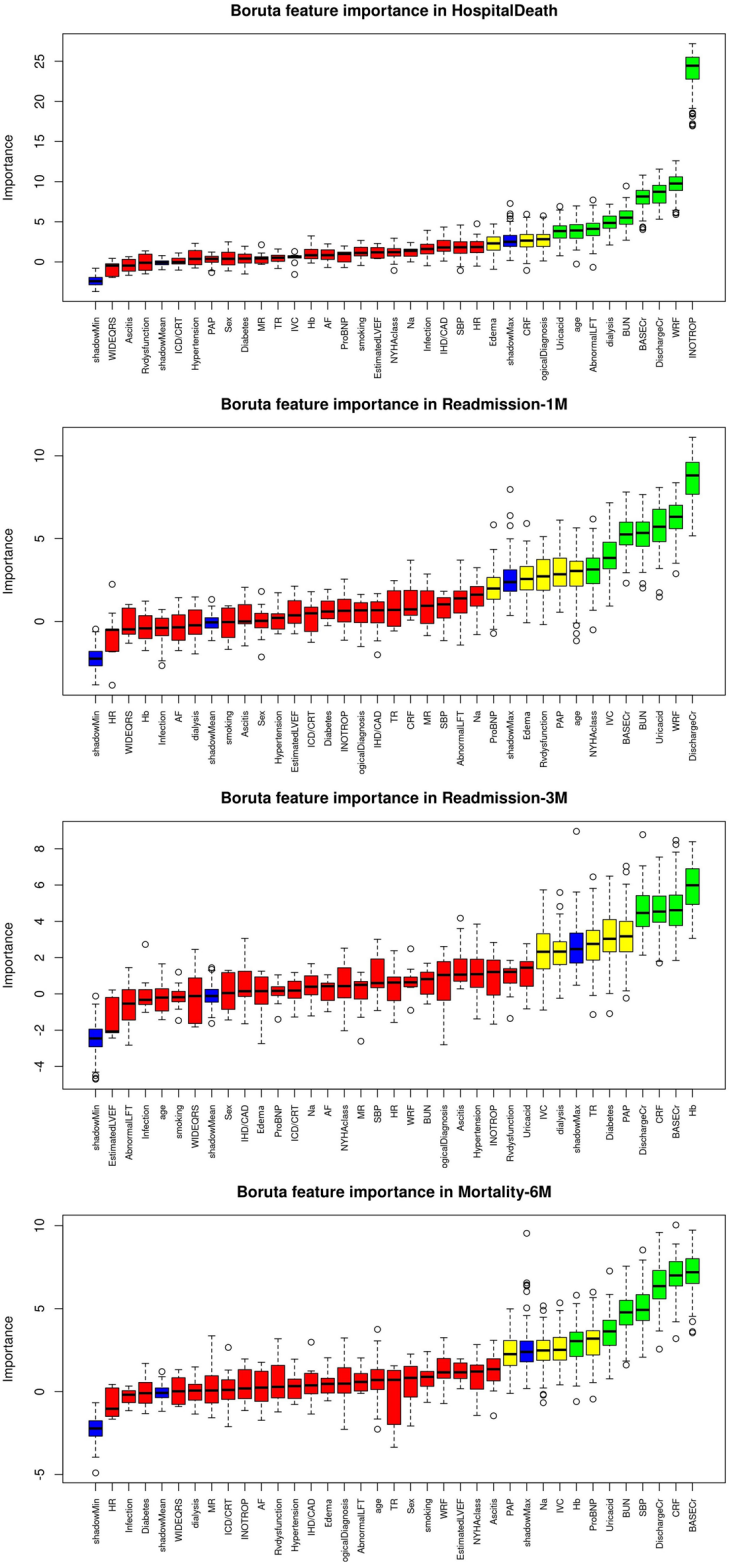
Boruta’s feature selection method depicts the importance of different features in four different outcomes: In-hospital mortality, 30-day hospital readmission, 3-month hospital readmission, and 6-month mortality, shown from top to bottom, respectively. Green, yellow and red colors indicate the importance of the selected features in order from more important to less important. Green, yellow, and red boxes represent important, tentative, and unimportant features and blue boxes represent shadow. Each boxplot has a minimal, average, and maximum amount of importance.

**Figure 3:**
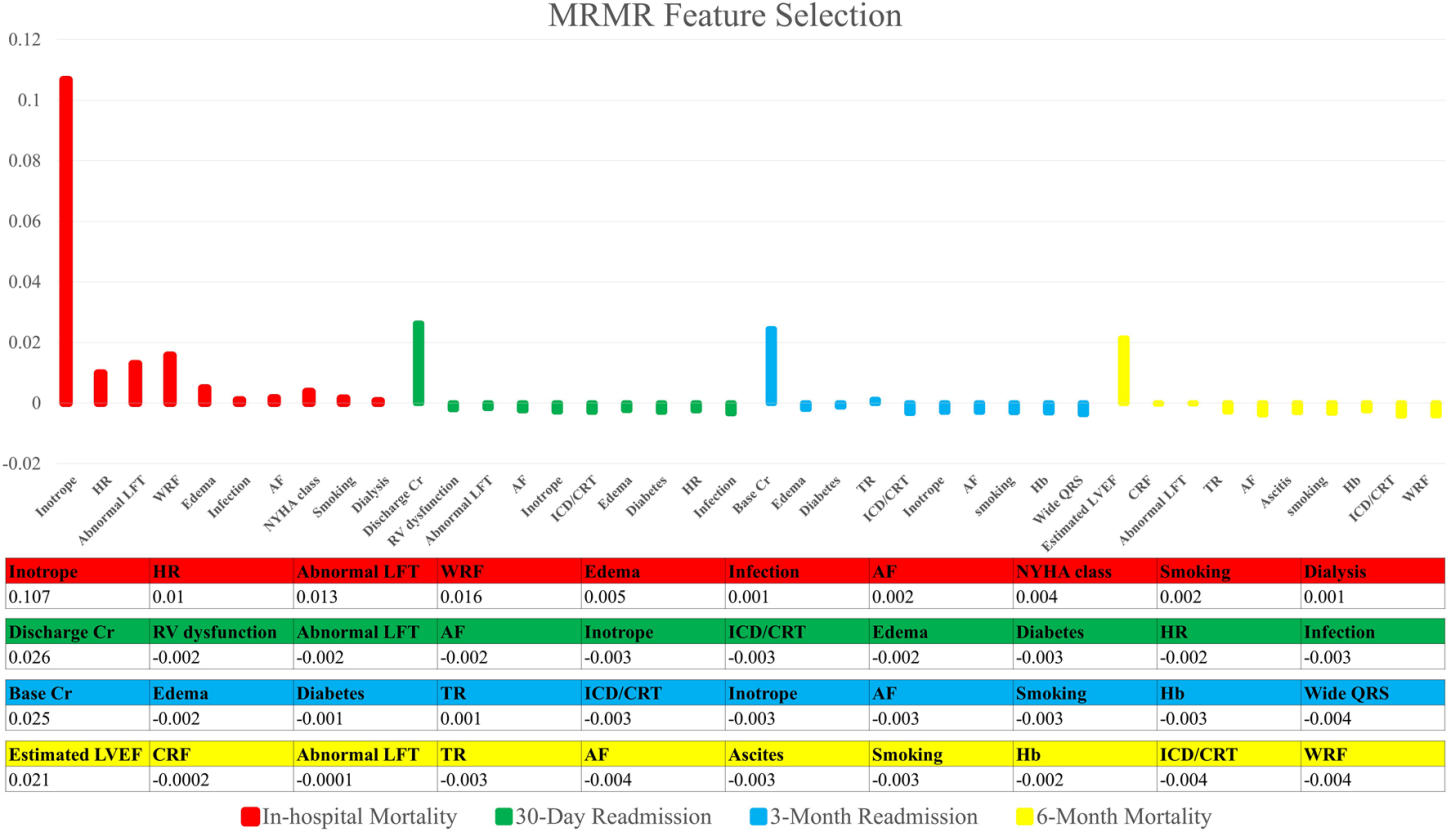
Features were ranked according to their importance values regarding four different outcomes according to MRMR’s feature selection. In-hospital mortality, 30-day hospital readmission, 3-month hospital readmission, and 6-month mortality are shown from left to right. The selected features are listed with their scores in the table under the figure.

**Figure 4:**
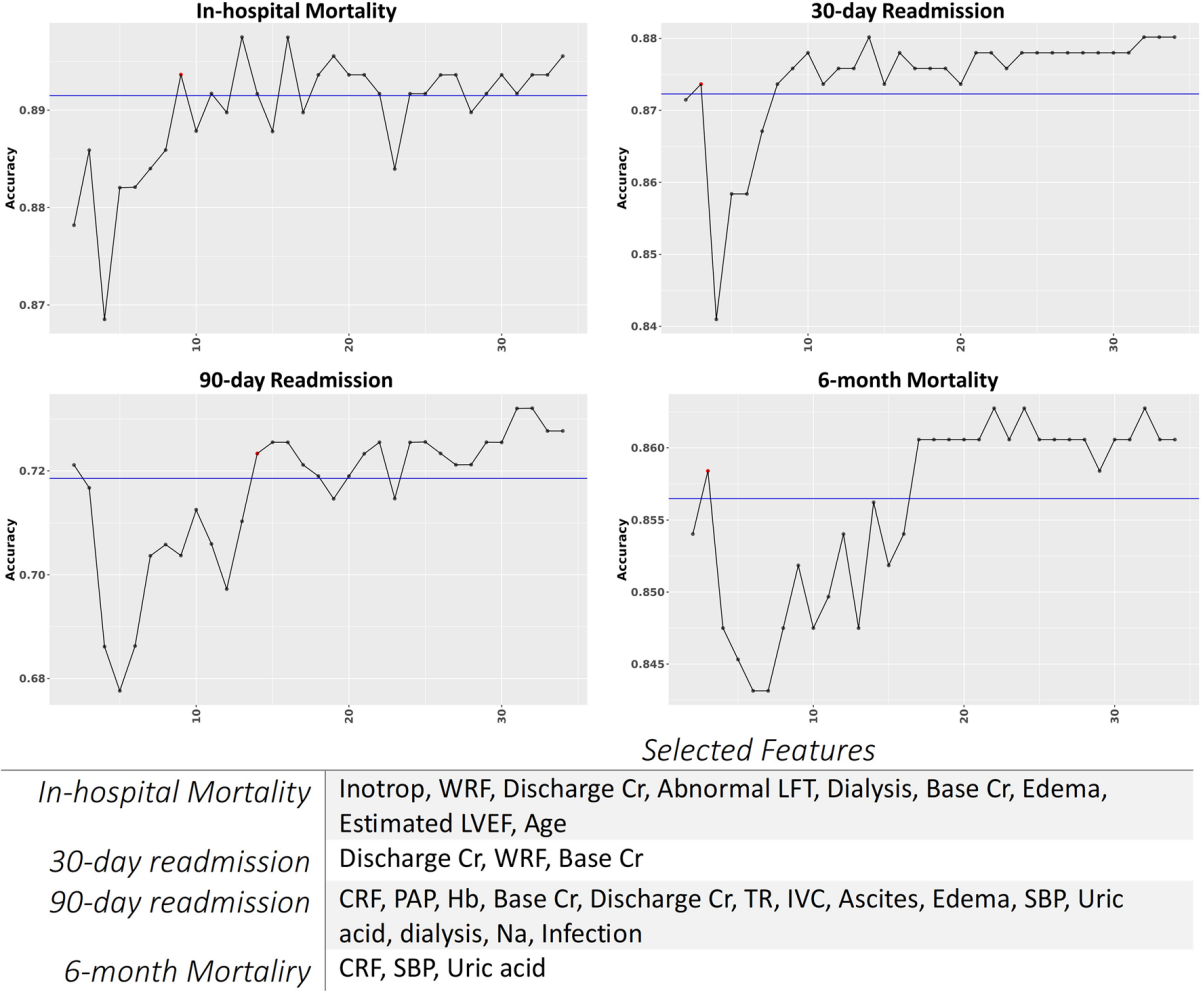
Illustration of RFE feature selection according to accuracy with 2 to 34 sets of features for four endpoints.

As mentioned before, this study used three different methods to select features: MRMR, Boruta, and RFE. As the best predictive features, Boruta’s method selected 12, 12, 9, and 11 features for in-hospital mortality, 30-day hospital readmission, 3-month hospital readmission, and 6-month mortality, respectively (Fig. 2). The MRMR analysis selected 10 features for each endpoint (Fig. 3). RFE feature selection chose 9, 3, 14, and 3 features for in-hospital mortality, 30-day readmission, 3-month readmission, and 6-month mortality, respectively (Fig. 4).

**Figure 5:**
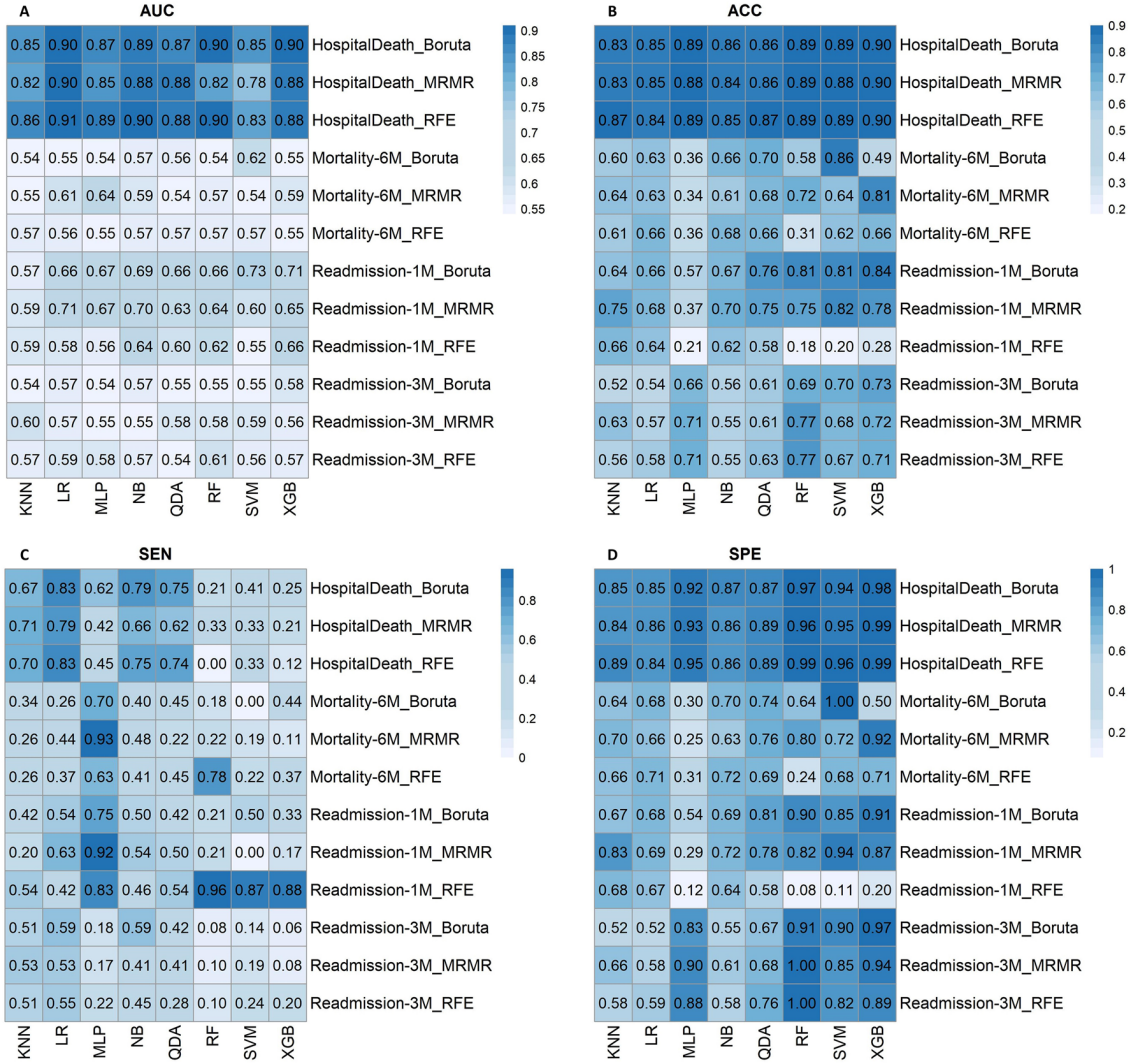
Illustration of the AUC (**A**), accuracy (**B**), sensitivity (**C**), and specificity (**D**) of four outcomes (in-hospital mortality, 6-month mortality, 30-day hospital readmission, and 3-month hospital readmission) using three different feature selection methods and eight classifiers.

**Figure 6:**
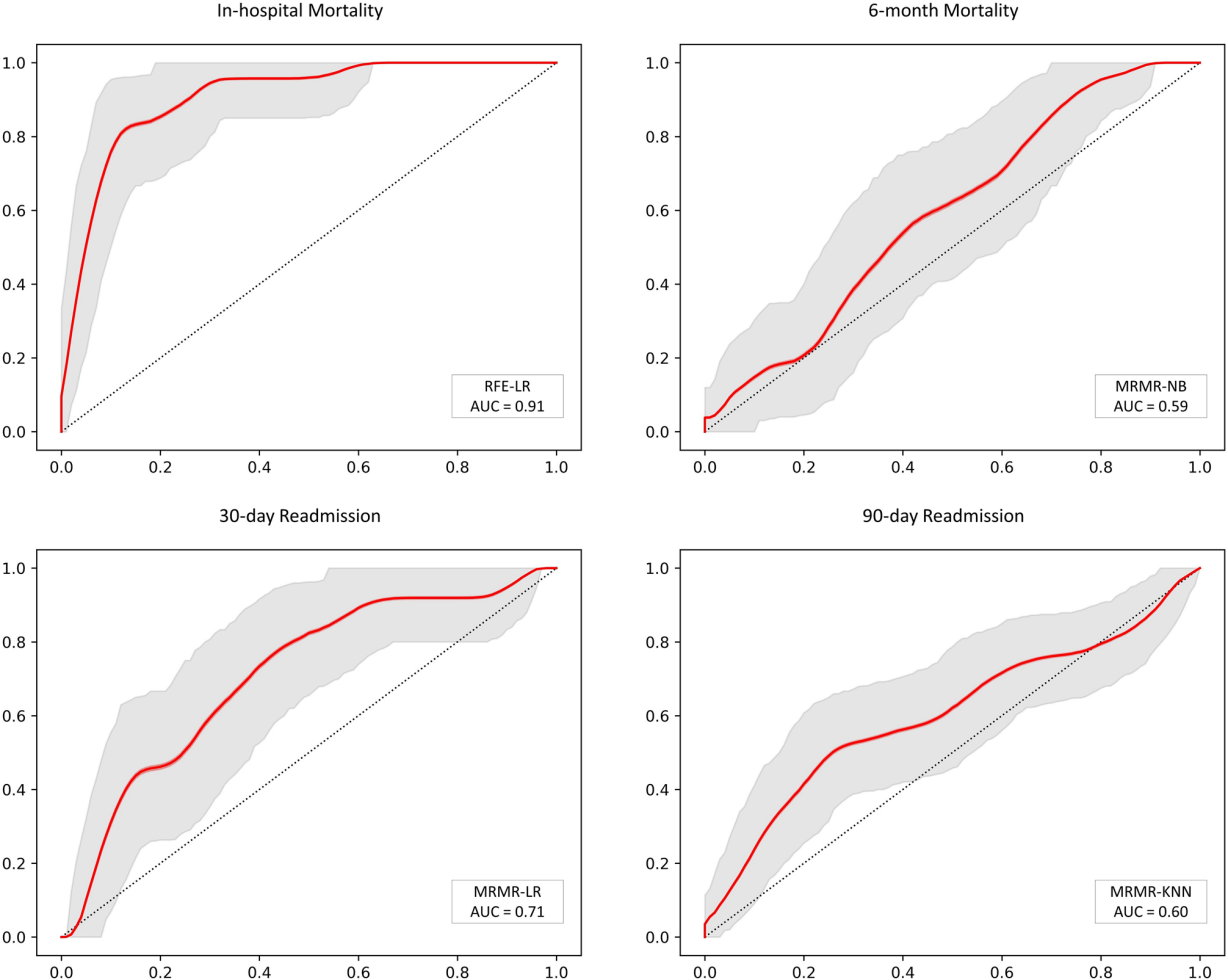
ROC curves of 4 best models in each endpoint.

Below are illustrations of performance metrics for each of the four outcomes. Eight classifiers and three feature selection methods were used and AUC, ACC, SEN, and SPE of each model were reported. The results of all the models of this study can be seen in Fig. 5, and the ROC curves of the best models from each endpoint can be seen in Fig. 6. Also, in Tables 2S–9S, all models’ mean, standard deviation, and confidence interval are given.

### In-hospital mortality

As represented in Fig. 5, several models performed well concerning in-hospital mortality. The RFE-LR (AUC: 0.91, ACC: 0.84, SPE: 0.84, SEN: 0.83) and Boruta-LR (AUC: 0.90, ACC: 0.85, SPE: 0.85, SEN: 0.83) models yield the best performance in terms of in-hospital mortality outcome prediction.

### 30-day readmission

Boruta-SVM and MRMR-LR models yield the best results for 30-day rehospitalization with AUC: 0.73 (ACC: 0.81, SPE: 0.85, SEN: 0.50) and AUC: 0.71 (ACC: 0.68, SPE: 0.69, SEN: 0.63), respectively.

### 3-month readmission

MRMR-KNN (AUC: 0.60, ACC: 0.63, SPE: 0.66, SEN: 0.53) was the top model for 3-month rehospitalization prediction.

### 6-month mortality

The MRMR-LR (AUC: 0.61, ACC: 0.63, SPE: 0.44, SEN: 0.66) and MRMR-NB (AUC: 0.59, ACC: 0.61, SPE: 0.48, SEN: 0.63) models performed better than others in terms of 6-month mortality prediction.

### Wilcoxon rank sum test

**Figure 7:**
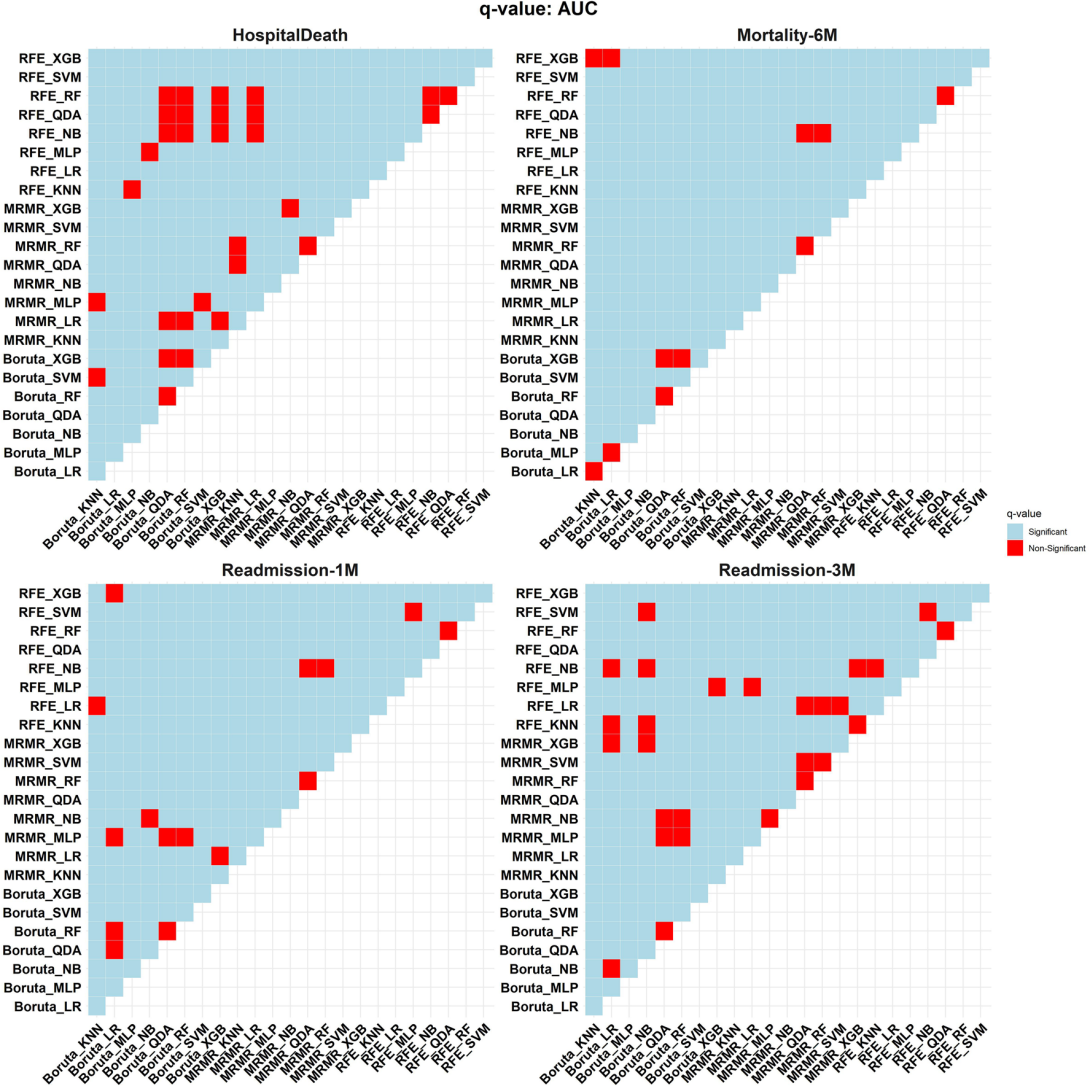
Wilcoxon signed-rank test is used to compare the performance of all models. Models were evaluated against each other in rows and columns. If the comparison between the row and column models shows a non-significant value, it would be highlighted in red. Light blue indicates that the column model was superior to the row model in terms of the p-value.

Using the Wilcoxon rank sum test, the AUC of different models in each endpoint was compared with all other 23 models. The overview of this comparison is given in Fig. 7. In the in-hospital mortality endpoint, RFE-LR, Boruta-LR, MRMR-LR, and RFE-NB as of best models in this end-point had 23, 23, 17, and 17 significant q-values, respectively. The best models in the 30-day rehospitalization endpoint were Boruta-SVM, MRMR-LR, MRMR-NB, and Boruta-NB with 23, 22, 22, and 22 significant q-values. The top model in the 3-month rehospitalization endpoint was the MRMR-KNN with 23 significant q-values. Finally, in the six-month mortality endpoint, the best models were MRMR-LR and MRMR-NB with 23 significant q-values.

## Discussion

In this study, ML frameworks were implemented for in-hospital mortality, 6-month mortality, 30-day hospital readmission, and 3-month hospital readmission prediction. Eight different classifiers and 34 features were used to predict different endpoints. The accurate readmission prediction may allow hospitals to focus on those who are at the highest risk. Feature selection in the in-hospital mortality led to the selection of Inotrop, WRF, Abnormal LFT, Dialysis, and Edema features in all three feature selection methods. Also, Discharge Cr, Base Cr, and age were selected by two feature selection methods. In the 30-day readmission, WRF, Base Cr, RV dysfunction, and Edema features were selected according to their importance in all feature selection methods, and Discharge Cr was also selected twice. In the 3-month readmission, the important features that were selected in all feature selection methods included Hb, Base Cr, and TR, and the opposite features were also selected twice: CRF, PAP, Discharge Cr, IVC, Edema, Dialysis, and Diabetes. Finally, CRF was selected three times, and SBP, Uric acid and Hb features were selected twice for 6-month mortality.

In patients with cardiovascular disease, kidney function evaluation is crucial due to its significant impact on patient outcomes. BUN and creatinine are usually used to evaluate kidney function, reflecting the kidneys’ ability to filter waste from the blood. Kidney function holds prominence as a key endpoint in various cardiovascular studies, cohorts, and registries^41^. Furthermore, Inotropes are prescribed for patients with low cardiac output. Epidemiological research demonstrates that within this specific group, the mortality rate is the highest^42^. Also, HF patients may be irresponsive to drug therapy and suffer from deteriorating functions of multiple organs, such as the kidney and liver^42^. Therefore, these characteristics affect the chance of readmissions or mortality more significantly^42^. Accordingly, in the study conducted by Luo et al*.*^24^, they included the liver functions of the patients by measuring minimum and mean values for Prothrombin Time (PT) and Partial Thromboplastin Time (PPT). In other studies by Li et al.^23^ and Kwon et al.^43^, renal functionality is assessed by measuring either means of creatinine or chronic renal insufficiency. In all mentioned studies, systolic and diastolic blood pressures were considered along with BUN. Kwon et al.^44^ also took EF, Left Ventricular Systolic Dimension (LVSD), Left Ventricular Diastolic Dimension (LVDD), and AF into account. In our study, we included any malfunctions in any liver tests to representing hepatic function by introducing Abnormal LFT feature. Regarding the kidney, Baseline Cr, Discharge Cr, WRF, dialysis, and CRF describe patients’ renal function.

We implemented three methods of selecting the features in this study: RFE, MRMR, and Boruta. Aligned with the previously mentioned important features, MRMR and Brouta feature selection methods scored Inotrope and WRF as the first and second significant features, respectively. Consequently, these two features are of great importance in predicting in-hospital mortality. The RFE feature selection method, however, does not provide the importance of each selected feature separately. As can be seen from the results, the best performance was achieved in the in-hospital mortality endpoint.

In the following sections, we will discuss different studies for various endpoints. Additionally, Table 2 compares the results of other studies with ours.

**Table 2:** A general comparison at one glance. Empty cells represent the information not reported by the authors.

Model	Endpoint	Cause	References	AUC	ACC	SEN	SPE
Deep neural network	In-hospital mortality	HF	Kwon et al.^44^	0.913	–	–	–
RFE-logistic regression		HF	This study	0.91	0.84	0.83	0.84
Boruta-logistic regression		HF	This study	0.90	0.85	0.83	0.85
MRMR-logistic regression		HF	This study	0.90	0.85	0.79	0.86
RFE-Naïve Bayes		HF	This study	0.90	0.85	0.75	0.86
Boruta-Naïve Bayes		HF	This study	0.89	0.86	0.79	0.87
Gradient boosting		HF	König et al.^22^	0.882	–	–	–
eXtreme gradient boosting		HF	König et al.^22^	0.882	–	–	–
RFE-quadratic discriminant analysis		HF	This study	0.88	0.87	0.74	0.89
MRMR-Naïve Bayes		HF	This study	0.88	0.84	0.66	0.86
MRMR-quadratic discriminant analysis		HF	This study	0.88	0.86	0.62	0.89
Deep neural network		HF	Kwon et al.^43^	0.88	–	–	–
Boruta-quadratic discriminant analysis		HF	This study	0.87	0.86	0.75	0.87
Boruta-multi-layer perceptron		HF	This study	0.87	0.89	0.62	0.92
RFE-K-nearest neighbors		HF	This study	0.86	0.87	0.70	0.89
LASSO regression		HF	Li et al.^23^	0.856	–	–	–
Boruta-K-Nearest neighbors		HF	This study	0.85	0.83	0.67	0.85
eXtreme gradient boosting		HF	Li et al.^23^	0.842	–	–	–
eXtreme gradient boosting		HF	Luo et al.^24^	0.831	–	–	–
MRMR-K-nearest neighbors		HF	This study	0.82	0.83	0.71	0.84
Boruta-support vector machine	30-day readmission	HF	This study	0.73	0.81	0.50	0.85
MRMR-logistic regression		HF	This study	0.71	0.68	0.63	0.69
Deep unified network		HF	Golas et al.^27^	0.705	–	–	–
MRMR-Naïve Bayes		HF	This study	0.70	0.70	0.54	0.72
Boruta-Naïve Bayes		HF	This study	0.69	0.67	0.50	0.69
Neural network		HF	Futoma et al.^45^	0.676	–	–	–
Multi-layer perceptron		HF	Awan et al.^26^	0.62	–	0.4842	0.7001
Neural network		HF	Liu et al.^29^	0.618	–	–	–
Support vector machine		HF	Zheng et al.^34^	–	0.784	0.973	0.086
Random forest		HF	Zheng et al.^34^	–	0.744	0.874	0.307
Logistic regression	3-month readmission	HF	Park et al.^31^	0.77	–	–	–
ML-LASSO + logistic regression		HF	Sarijaloo et al.^35^	0.76	–	0.83	–
Logistic regression		HF	Park et al.^31^	0.755	–	–	–
MRMR-K-Nearest neighbors		HF	This study	0.60	0.63	0.53	0.66
MRMR-logistic regression	6-month mortality	HF	This study	0.61	0.63	0.44	0.66
MRMR-Naïve Bayes		HF	This study	0.59	0.61	0.48	0.63

### In-hospital mortality

Luo et al.^24^ designed an ML model using the clinical features to investigate in-hospital mortality risk from HF. They showed that ML models can predict in-hospital mortality risks better than conventional methods. Their best results were obtained from XGB with AUC: 0.831 and 0.809 in internal and external datasets, respectively. In^22^, five ML algorithms were used to develop models for predicting in-hospital mortality due to HF in 59,125 cases with 6.2% in-hospital mortality. The best-performing algorithms among all five models were XGB and GB with AUC: 0.882. Li et al.^23^ investigated in-hospital deaths due to HF in 1177 patients, among which 13.52% died in the hospital. The LASSO regression model outperformed XGB and GWTG-HF risk scores with an AUC of 0.8562 (AUC via bootstrap: 0.8518). Kwon et al.^43^ used the deep neural network (DNN) method for predicting the mortality of patients with AHF (DAHF). A total of 12,644 datasets from two hospitals were utilized as train datasets and 4759 datasets from 10 hospitals were used as test datasets. The DAHF method demonstrated the highest performance with an AUC of 0.880. In another study, Kwon et al.^44^ used a deep learning model to investigate the in-hospital mortality of patients with heart disease based on their echocardiography. In total, 25,776 patients from two hospitals were enrolled. The second hospital (external dataset) is also divided into two subgroups: Coronary Artery Disease (CAD) and HF. The model with AUC: 0.912, 0.898, 0.958, and 0.913 for internal and external validation, CAD, and HF had the best performance, respectively. Our models also demonstrated reliable performance like the models reported in^22–24,43^. Notably, our study provided all four evaluation metrics (AUC, ACC, SEN, SPE) with promising values, while in some studies, AUC is solely reported, which is not a suitable metric in the unbalanced dataset.

### 30-day readmission

In a study by Liu et al.^29^, 303,233 patients hospitalized with congestive HF were investigated for unplanned 30-day readmissions. Of them, 53,649 cases were readmitted after 30 days. The Medical Code Embedding Deep Set Architecture (MCEDSA) model showed the highest result with an AUC of 0.618. Awan et al.^26^ examined HF readmissions or deaths within 30 days in 10,757 patients, of which 23.6% of cases died or were readmitted after 30 days. They obtained an AUC of 0.62 (Sensitivity: 48.42%, Specificity: 70.01%) by the MLP model. Zheng et al.^34^ implemented models for 1641 HF patients (316 instances were re-hospitalized 30 days after discharge). They also compared the results with the LACE score (L: Length of patient stay in the hospital, A: Acuity of admission of the patient in the hospital, C: Comorbidity, and E: Emergency visit) risk-prediction model. Due to imbalanced data, they utilized a replication-based random oversampling technique. As a result, they achieved an accuracy of 78.4% (Sensitivity: 97.3%, Specificity: 8.6%) for PSO-SVM with Radial Basis Function (RBF) as their best model. Futoma et al.^45^ used an MLP to predict 30-day hospital readmission, in which 1,328,384 patients with various diseases were included, with 19% of cases of HF being readmitted in 30 days, which had an AUC of 0.676. However, they only provided an AUC metric, which may not be considered an adequate performance metric for imbalanced datasets. In another study, Golas et al.^27^ examined 30-day readmission risk prediction on 11,510 unique HF patients (6,369 cases were readmitted within 30 days after discharge). They trained a Deep Unified Network (DUN) model and compared it to LR, GB, and maxout networks. Our study’s best-developed model in the 30-day readmission endpoint was Boruta-SVM (AUC: 0.71, ACC: 0.68, SEN: 0.63, and SPE: 0.69).

### 3-month readmission

In the study of Sarijaloo et al.^35^, SVM, RF, LASSO, and GB ML models were used to select the best features engaged with 90-day readmissions in 3189 HF patients, with 15.2% 90-day readmission. Extracted variables were fed to the LR algorithm to develop the risk-predictor model to evaluate 90-day readmissions and deaths. Results showed that the ML-LASSO + LR model, with an AUC and SEN of 0.760 and 0.83, was superior to all other models. Park et al.^31^ evaluated 90-day rehospitalization and death of patients with HF by dividing the dataset into two groups: 1965 sufferers of HF with preserved EF (HFpEF) and 1124 individuals suffering HF with reduced EF (HFrEF). The reported average AUC for each model showed that the HFpEF model, with an AUC of 0.770, performed better than both generic and HFrEF models. Our best model’s performance in this endpoint was MRMR-KNN (AUC: 0.60, ACC: 0.63, SEN: 0.53, and SPE: 0.66) was not satisfying. However, it is worth noting that most of the other studies typically reported AUC alone, which restricts the comprehensive evaluation of a model.

### 6-month mortality

To the best of our knowledge, no ML study has addressed the 6-month mortality prediction. Our study yielded suboptimal results; nevertheless, it serves as an initial point for future research in 6-month mortality prediction. In conclusion, short-term endpoints (in-hospital mortality and 30-day readmission) exhibited better performance than long-term endpoints (3-month readmission and 6-month mortality), possibly due to the relevance of features used in this study to short-term outcomes. However, further investigations are warranted to confirm this observation.

## Limitations

Our study suffers from a few limitations concerning research methodology. The number of samples we examined was small compared to other studies. So, we used different feature selection methods, stratified tenfold cross-validation for hyperparameter optimization, and bootstrapping on test data to avoid the overfitting problem. Of 737 patients, 529 were male (71%), which makes up a disproportionate patient population compared to 208 female patients (29%). Moreover, single-centered data collection may influence the results by particular socioeconomic or demographic aspects. In addition, some clinical features used in other studies could not be collected in this research. Other variables could contribute to the predictability, but their inclusion would require more time and expense. We did not consider non-clinical factors such as psychological support despite the importance^28^. In addition, quality of life and functionality should be considered as endpoints, but this study only focused on mortality and readmissions. Finally, the lack of external data was also a limitation of the study. To sum up, in the future, there is potential to further improve the development of machine learning models by using more extensive and diverse datasets. This can include collecting data from multiple centres or sources, which can help to increase the generalizability of the models across different populations and contexts. In addition, deep learning methods have shown great promise in recent years and can be utilized to further enhance the performance of machine learning models.

## Conclusion

This study highlighted the potential use of ML in predicting patients with HF who experienced in-hospital mortality and 30-day rehospitalization. Implementing conventional features and ML methods lead to reliable predictive models to predict patients who experienced in-hospital mortality and 30-day rehospitalization. These models could potentially help physicians thoroughly examine specific patients and target more effective hospital care. They also can be potentially effective in personalizing treatment, decision-making, and better budget allocation. However, the models designed to predict 3-month rehospitalization and 6-month mortality in this study need more investigation. Therefore, more studies must be conducted to achieve more promising results in other endpoints.

### Supplementary Information

Supplementary Information.

## Data Availability

The datasets used/analyzed in the current study are available from corresponding authors on request.

## References

[1] James SL (2018). Global, regional, and national incidence, prevalence, and years lived with disability for 354 diseases and injuries for 195 countries and territories, 1990–2017: A systematic analysis for the Global Burden of Disease Study 2017. Lancet.

[2] Sinnenberg L, Givertz MM (2020). Acute heart failure. Trends Cardiovasc. Med..

[3] Arrigo M (2020). Acute heart failure. Nat. Rev. Dis. Primers.

[4] Komanduri S, Jadhao Y, Guduru SS, Cheriyath P, Wert Y (2017). Prevalence and risk factors of heart failure in the USA: NHANES 2013–2014 epidemiological follow-up study. J. Commun. Hosp. Internal Med. Perspect..

[5] Safiriyu IA, Asemota IR, Akuna E, Ehizogie E (2022). The impact of acute heart failure related length of stay on the 30-day all-cause readmission rate. J. Cardiac. Fail..

[6] Lan T (2021). Mortality and readmission rates after heart failure: A systematic review and meta-analysis. Ther. Clin. Risk Manag..

[7] Khan, M. S. *et al.* Trends in 30-and 90-day readmission rates for heart failure. *Circulation: Heart Failure* **14**, e008335 (2021). 10.1161/CIRCHEARTFAILURE.121.008335 33866827

[8] Fudim, M., *et al.* Aetiology, timing and clinical predictors of early vs. late readmission following index hospitalization for acute heart failure: Insights from ASCEND‐HF. *Eur. J. Heart Fail.* **20**, 304–314 (2018). 10.1002/ejhf.1020 PMC5826892 29082629

[9] Urbich M (2020). A systematic review of medical costs associated with heart failure in the USA (2014–2020). PharmacoEconomics.

[10] Lahewala, S. *et al.* Heart failure: same-hospital vs. different-hospital readmission outcomes. *Int. J. Cardiol.* **278**, 186–191 (2019). 10.1016/j.ijcard.2018.12.043 30579719

[11] Fingar, K. & Washington, R. Trends in hospital readmissions for four high-volume conditions, 2009–2013: statistical brief# 196. (2016). 26764446

[12] Yu S (2015). Predicting readmission risk with institution-specific prediction models. Artif. Intell. Med..

[13] Minott J (2008). Reducing hospital readmissions. Acad. Health.

[14] Lee KK, Yang J, Hernandez AF, Steimle AE, Go AS (2016). Post-discharge follow-up characteristics associated with 30-day readmission after heart failure hospitalization. Med. Care.

[15] Wan TT (2017). Strategies to modify the risk of heart failure readmission: A systematic review and meta-analysis. Health Serv. Res. Manag. Epidemiol..

[16] Vader JM (2016). Timing and causes of readmission after acute heart failure hospitalization-insights from the heart failure network trials. J. Card. Fail..

[17] Mirkin KA, Enomoto LM, Caputo GM, Hollenbeak CS (2017). Risk factors for 30-day readmission in patients with congestive heart failure. Heart Lung.

[18] Natale, J. & Wang, S. in *IIE Annual Conference. Proceedings.* 3518 (Institute of Industrial and Systems Engineers (IISE)).

[19] Hosseinzadeh, A., Izadi, M., Verma, A., Precup, D. & Buckeridge, D. in *Twenty-fifth IAAI conference.*

[20] Jiang S, Chin K-S, Qu G, Tsui KL (2018). An integrated machine learning framework for hospital readmission prediction. Knowl. Based Syst..

[21] Hosseinzadeh, A. Mining hospital admission-discharge data to discover the chance of readmission. (2013).

[22] König S (2021). Machine learning algorithms for claims data-based prediction of in-hospital mortality in patients with heart failure. ESC Heart Fail..

[23] Li F (2021). Prediction model of in-hospital mortality in intensive care unit patients with heart failure: Machine learning-based, retrospective analysis of the MIMIC-III database. BMJ Open.

[24] Luo C (2022). A machine learning-based risk stratification tool for in-hospital mortality of intensive care unit patients with heart failure. J. Transl. Med..

[25] Allam A, Nagy M, Thoma G, Krauthammer M (2019). Neural networks versus Logistic regression for 30 days all-cause readmission prediction. Sci. Rep..

[26] Awan SE, Bennamoun M, Sohel F, Sanfilippo FM, Dwivedi G (2019). Machine learning-based prediction of heart failure readmission or death: Implications of choosing the right model and the right metrics. ESC Heart Fail..

[27] Golas SB (2018). A machine learning model to predict the risk of 30-day readmissions in patients with heart failure: A retrospective analysis of electronic medical records data. BMC Med. Inf. Decis. Mak..

[28] Krumholz HM (1998). Prognostic importance of emotional support for elderly patients hospitalized with heart failure. Circulation.

[29] Liu W (2020). Predicting 30-day hospital readmissions using artificial neural networks with medical code embedding. PloS One.

[30] Mahajan, S. M. & Ghani, R. in *Medinfo.* 243–247.

[31] Park, J., Zhong, X., Babaie Sarijaloo, F. & Wokhlu, A. Tailored risk assessment of 90‐day acute heart failure readmission or all‐cause death to heart failure with preserved versus reduced ejection fraction. *Clin. Cardiol.* **45**, 370–378 (2022). 10.1002/clc.23780 PMC9019897 35077583

[32] Rahimi AR, Spertus JA, Reid KJ, Bernheim SM, Krumholz HM (2007). Financial barriers to health care and outcomes after acute myocardial infarction. JAMA.

[33] Shin S (2021). Machine learning vs. conventional statistical models for predicting heart failure readmission and mortality. ESC Heart Fail..

[34] Zheng B (2015). Predictive modeling of hospital readmissions using metaheuristics and data mining. Expert Syst. Appl..

[35] Sarijaloo F, Park J, Zhong X, Wokhlu A (2021). Predicting 90 day acute heart failure readmission and death using machine learning-supported decision analysis. Clin. Cardiol..

[36] Naderi N (2022). Predictors of readmission in hospitalized heart failure patients. J. Cardiovasc. Thorac. Res..

[37] McDonagh TA (2021). 2021 ESC Guidelines for the diagnosis and treatment of acute and chronic heart failure: Developed by the Task Force for the diagnosis and treatment of acute and chronic heart failure of the European Society of Cardiology (ESC) With the special contribution of the Heart Failure Association (HFA) of the ESC. Eur. Heart J..

[38] UK, N. A.-A. *et al.* 2016 ESC Guidelines for the diagnosis and treatment of acute and chronic heart failure. *Eur. Heart J.* **37**, 2129–2200 (2016). 10.1093/eurheartj/ehw128 27206819

[39] Tsuchihashi M (2001). Medical and socioenvironmental predictors of hospital readmission in patients with congestive heart failure. Am. Heart J..

[40] Bischl B (2016). mlr: Machine learning in R. J. Mach. Learn. Res..

[41] Lopez-Giacoman S, Madero M (2015). Biomarkers in chronic kidney disease, from kidney function to kidney damage. World J. Nephrol..

[42] Farmakis D (2019). A pragmatic approach to the use of inotropes for the management of acute and advanced heart failure: An expert panel consensus. Int. J. Cardiol..

[43] Kwon J-M (2019). Artificial intelligence algorithm for predicting mortality of patients with acute heart failure. PloS One.

[44] Kwon JM, Kim KH, Jeon KH, Park J (2019). Deep learning for predicting in-hospital mortality among heart disease patients based on echocardiography. Echocardiography.

[45] Futoma J, Morris J, Lucas J (2015). A comparison of models for predicting early hospital readmissions. J. Biomed. Inf..

