# Supplementary material for: Machine learning based readmission and mortality prediction in heart failure patients
Source: Sci Rep. 2023 Oct 31;13:18671. doi: 10.1038/s41598-023-45925-3 (PMC10618467; doi:10.1038/s41598-023-45925-3)
Supplement: Supplementary file 1 — Supplementary Information. [file 41598_2023_45925_MOESM1_ESM.docx]

**Table 1S.** The value of the features implemented in this study.

|  | | In-Hospital Mortality | 30-Day Readmission | 3-Month Readmission | 6-Month Mortality | No Event |
| --- | --- | --- | --- | --- | --- | --- |
| Continuous features | Case  Feature | **82** | **81** | **170** | **91** | **433** |
|  | Age | **58.24±17.65** | **52.36±17.00** | **55.17±16.65** | **59.01±16.43** | **55.53±17.44** |
|  | Heart Rate | **89.71±20.14** | **87.05±20.69** | **87.17±21.90** | **84.93±18.74** | **87.21±20.15** |
|  | SBP | **97.20±18.95** | **106.16±22.34** | **108.75±25.29** | **108.54±21.89** | **113.67±26.16** |
|  | Base Cr | **1.80± 1.19** | **1.72±0.90** | **1.75±1.26** | **1.85±1.46** | **1.52±1** |
|  | BUN | **45.00±32.41** | **37.70±26.85** | **36.06±27.52** | **39.65±27.70** | **33.42±25.78** |
|  | Uric acid | **7.74±2.61** | **8.26±2.78** | **7.53±2.82** | **7.90±2.86** | **7.42±2.48** |
|  | Hb | **11.66±1.98** | **12.17±1.96** | **12.13±2.04** | **11.97±2.17** | **12.61±2.1** |
|  | Na | **133.72±5.30** | **135.03±14.80** | **135.07±10.91** | **135.66±5.43** | **135.59±5.03** |
|  | Pro BNP | **13832.63±11785.27** | **9374.43±8294.63** | **8104.65±8356.61** | **10546.65±9744.21** | **8127.30±8505.40** |
|  | Discharge Cr | **2.30±1.59** | **1.94±1.04** | **1.68±0.99** | **1.85±1.10** | **1.52±0.84** |
|  | LVEF | **18.02±7.74** | **17.47±6.69** | **19.07±7.69** | **17.12±7.05** | **19.24±8.78** |
|  | PAP | **48.33±16.28** | **48.60±16.00** | **47.65±15.47** | **45.69±16.66** | **42.79±14.35** |
|  | IVC | **21.97±7.77** | **22.39±7.36** | **21.09±7.64** | **20.98±8.13** | **19.18±7.56** |
| Categorical features | Sex | **60 males+**  **22 females** | **60 males+**  **21 females** | **126 males+**  **44 females** | **76 males+**  **15 females** | **326 males+**  **107 females** |
|  | AHF etiology | **42 Ischemic+**  **40 non-ischemic** | **39 Ischemic+**  **42 non-ischemic** | **89 Ischemic+**  **81 non-ischemic** | **56 Ischemic+**  **35 non-ischemic** | **199 Ischemic+**  **234 non-ischemic** |
|  | Diabetes Mellitus | **25** | **23** | **54** | **33** | **178** |
|  | Hyper tension | **25** | **28** | **60** | **30** | **181** |
|  | IHD | **41** | **39** | **88** | **55** | **233** |
|  | CKD | **36** | **39** | **69** | **43** | **133** |
|  | Smoking | **17** | **27** | **56** | **35** | **140** |
|  | ICD | **21** | **27** | **45** | **23** | **110** |
|  | Edema | **43** | **36** | **66** | **40** | **123** |
|  | Ascites | **39** | **26** | **49** | **28** | **93** |
|  | Infection | **54** | **45** | **73** | **46** | **184** |
|  | Dialysis | **20** | **6** | **14** | **9** | **27** |
|  | Inotropes usage | **74** | **28** | **42** | **25** | **96** |
|  | Wide QRS | **40** | **31** | **53** | **33** | **174** |
|  | Atrial Fibrillation | **34** | **20** | **38** | **20** | **127** |
|  | Abnormal LFT | **54** | **36** | **61** | **40** | **146** |
|  | WRF | **60** | **41** | **61** | **38** | **144** |
|  | RV dysfunction | **43** | **50** | **87** | **50** | **232** |
|  | MR | **54** | **49** | **97** | **49** | **221** |
|  | TR | **57** | **54** | **109** | **57** | **256** |
|  | NYHA class | **1 : 0 2 : 20**  **2.5 : 1 3 : 29**  **3.5 : 8 4 : 24** | **1 : 4 2 : 15**  **2.5 : 1 3 : 36**  **3.5 : 4 4 : 21** | **1 : 5 2 : 27**  **2.5 : 5 3 : 93**  **3.5 : 10 4 : 30** | **1 : 4 2 : 14**  **2.5 : 3 3 : 54**  **3.5 : 1 4 : 15** | **1 : 8 2 : 74**  **2.5 : 18 3 : 257**  **3.5 : 37 4 : 80** |

**Feature Selection Methods**

In this section, we present a comprehensive analysis of the feature selection techniques used in our study. Feature selection plays a crucial role in identifying the most informative variables and improving the performance and interpretability of our models. We employed three prominent methods, namely Recursive Feature Elimination (RFE), Minimum Redundancy Maximum Relevance (MRMR), and Boruta. The following subsections elaborate on each method's principles.

**Recursive Feature Elimination (RFE)**

Recursive Feature Elimination (RFE) ^1^ is a prominent feature selection technique employed in machine learning and data analysis. Its primary objective is to identify the most relevant features within a given dataset. RFE operates as a backward selection method, starting with all the features and iteratively removing the least important ones until the desired subset is obtained ^2^.

The RFE algorithm follows these steps:

Step 1: Model Training: Initially, a machine learning model is trained using all the features in the dataset.

Step 2: Feature Importance Assessment: Importance scores are assigned to each feature based on their impact on the model's performance (accuracy). Various methods can be used to evaluate feature importance, such as weights in a linear model or feature importance in a tree-based model.

Step 3: Recursive Elimination: In each iteration, RFE eliminates the feature(s) with the lowest importance score.

Step 4: Model Re-Training: After feature elimination, the model is re-trained using the reduced feature set.

Step 5: Iteration: Up until the desired number of features is reached or a stopping criterion is satisfied, steps 2 to 4 are iteratively repeated.

By repeatedly removing the least important features and retraining the model, RFE effectively identifies the subset of features that contributes most significantly to the model's performance. This process helps mitigate the risk of overfitting and improves the model's generalization capabilities ^3^.

**Minimum Redundancy Maximum Relevance (MRMR)**

The MRMR ^4^ algorithm operates in two steps: relevance calculation and redundancy reduction. In the relevance calculation step, each feature's relevance to the target variable is evaluated using an appropriate measure, such as mutual information (Equation 1) ^5^. Mutual information measures the dependence between two variables. While, if X and Z are independent random variables, their mutual information (I) is zero, indicating no statistical dependence or shared information between them. On the other hand, if X and Z represent the same random variable, the mutual information I equals the entropy of X, which measures the intrinsic uncertainty and information content of that variable. To achieve this, MRMR employs an iterative approach. In each iteration, it selects the feature with the highest relevance to the target variable (Equation 2) and adds it to the feature subset. Subsequently, it evaluates the redundancy between this selected feature (Equation 3) and the features already included in the subset. The feature that exhibits the lowest redundancy with the selected subset is then added. This process continues iteratively until the desired number of features is selected. According to below equations, the objective of MRMR algorithm is to identify an optimal feature set (S) that achieves two goals: maximizing the relevance of S to a response variable y (denoted as V_S_) and minimizing the redundancy within S (denoted as WS). The relevance and redundancy measures, V_S_ and W_S_, respectively, are quantified using mutual information I ^6^.

Equation 1 : *$I(X,Z)=\sum_{i,j}P(X=x_{i},Z=z_{j})log\frac{P(X=x_{i},Z=z_{j})}{P(X=x_{i})P(Z=z_{j})}$*

Where I is the entropy of X. X and Z are random variables.

Equation 2 : $V_{S}=\frac{1}{\mid S\mid}\sum_{x\in S}I(x,y)$

Where V_S_ is the entropy of a feature and the target. S is the feature set.

Equation 3 : *$W_{S}=\frac{1}{\mid S\mid^{2}}\sum_{x,z\in S}I(x,z)$*

Where W_S_ is the entropy of the two features ^7^.

**Boruta**

The Boruta ^8^ algorithm is a feature selection technique that aims to identify relevant features within a dataset. It is particularly advantageous in machine learning scenarios involving a large number of potential input variables or features, where the objective is to determine which features are most informative for accurate predictions. The algorithm operates through a series of iterations. Initially, each feature is assigned a tentative importance score based on a random forest model's evaluation metrics, such as accuracy. Subsequently, Boruta compares the importance of each feature to the maximum importance observed among its corresponding randomly generated shadow features. These shadow features are created by randomly permuting the values of the original features, effectively severing any underlying relationship with the target variable.

By comparing the importance of each real feature to that of its shadow counterparts, Boruta determines whether a feature is statistically significant or not. If a feature's importance significantly exceeds that of its shadows, it is considered a selected feature. The algorithm proceeds to the next iteration until all features are either confirmed or rejected. In each iteration, an original feature is compared to its corresponding shadow feature in terms of the model's performance. If the original feature exhibits higher importance than its shadow feature, it is selected. The count of times a feature is selected across all iterations is then tallied, allowing for the determination of each feature's relative importance. Features with higher counts are considered more important ^9^.

Table 2S. The results of different models in hospital death endpoint

Table 3S. The range of metrics in different models in hospital death endpoint

Table 4S. The results of different models in 6-month mortality endpoint

Table 5S. The range of metrics in different models in 6-month mortality endpoint

Table 6S. The results of different models in one-month readmission endpoint

Table 7S. The range of metrics in different models in one-month readmission endpoint

Table 8S. The results of different models in 3-month readmission endpoint

Table 9S. The range of metrics in different models in 3-month readmission endpoint

**References**

1 Guyon, I., Weston, J., Barnhill, S. & Vapnik, V. Gene selection for cancer classification using support vector machines. *Machine learning* **46**, 389-422 (2002).

2 Menze, B. H. *et al.* A comparison of random forest and its Gini importance with standard chemometric methods for the feature selection and classification of spectral data. *BMC bioinformatics* **10**, 1-16 (2009).

3 Sabouri, M. *et al.* Myocardial Perfusion SPECT Imaging Radiomic Features and Machine Learning Algorithms for Cardiac Contractile Pattern Recognition. *Journal of Digital Imaging* **36**, 497-509 (2023).

4 Mohebi, M. *et al.* Post-revascularization Ejection Fraction Prediction for Patients Undergoing Percutaneous Coronary Intervention Based on Myocardial Perfusion SPECT Imaging Radiomics: a Preliminary Machine Learning Study. *Journal of Digital Imaging*, 1-16 (2023).

5 Ding, C. & Peng, H. Minimum redundancy feature selection from microarray gene expression data. *Journal of bioinformatics and computational biology* **3**, 185-205 (2005).

6 Shiri, I. *et al.* Machine learning-based prognostic modeling using clinical data and quantitative radiomic features from chest CT images in COVID-19 patients. *Computers in biology and medicine* **132**, 104304 (2021).

7 Darbellay, G. A. & Vajda, I. Estimation of the information by an adaptive partitioning of the observation space. *IEEE Transactions on Information Theory* **45**, 1315-1321 (1999).

8 Kursa, M. B. & Rudnicki, W. R. Feature selection with the Boruta package. *Journal of statistical software* **36**, 1-13 (2010).

9 Chen, R.-C., Dewi, C., Huang, S.-W. & Caraka, R. E. Selecting critical features for data classification based on machine learning methods. *Journal of Big Data* **7**, 52 (2020).
